# Exploratory Multi-Level Analysis of the HIF Axis in Clear-Cell Renal Cell Carcinoma and Evaluation of GN44028 as an Experimental HIF Pathway-Modulating Compound

**DOI:** 10.3390/ijms27083505

**Published:** 2026-04-14

**Authors:** Piotr M. Wierzbicki, Agnieszka Rybarczyk, Mateusz Czajkowski, Jacek Kieżun, Bartłomiej E. Kraziński, Anna Olszewska, Marzena Kogut-Wierzbicka, Zuzanna Rudaś, Aleksandra Kierczak, Karol Mitas, Laura Wrońska, Michalina Grudzińska, Patrik da Silva Vital, Anna Kotulak-Chrząszcz

**Affiliations:** 1 Department of Histology, Faculty of Medicine, Medical University of Gdańsk, 80-210 Gdańsk, Poland; amazurek@gumed.edu.pl (A.R.); anna.olszewska@gumed.edu.pl (A.O.); annkot@gumed.edu.pl (A.K.-C.); 2 Department of Urology, Faculty of Medicine, Medical University of Gdańsk, 80-210 Gdańsk, Poland; mateusz.czajkowski@gumed.edu.pl; 3 Department of Anatomy and Histology, School of Medicine, Collegium Medicum, University of Warmia and Mazury, 10-709 Olsztyn, Polandbartlomiej.krazinski@uwm.edu.pl (B.E.K.); 4 Faculty of Medicine, Academy of Applied Medical and Sciences, 82-300 Elbląg, Poland; m.kogut-wierzbicka@amisns.edu.pl; 5 Student Scientific Circle, Department of Histology, Faculty of Medicine, Medical University of Gdańsk, 80-210 Gdańsk, Polandk.mitas@gumed.edu.pl (K.M.); laurawronska@gumed.edu.pl (L.W.); 6 Student Scientific Circle, Department of Urology, Faculty of Medicine, Medical University of Gdańsk, 80-210 Gdańsk, Poland; 7 Molecular Oncology Research Center, Pio XII Foundation, Barretos Cancer Hospital, Barretos 14780-360, SP, Brazil; patrikdasilvavital@gmail.com

**Keywords:** clear-cell renal cell carcinoma, GN44028, HIF1A, EPAS1/HIF2A, hypoxia-inducible factors, immunohistochemistry, TCGA, therapeutic resistance

## Abstract

Clear-cell renal cell carcinoma (ccRCC) is characterised by constitutive activation of hypoxia-inducible factors (HIFs) following VHL loss, which contributes to tumour progression and therapeutic resistance. Given the limitations of VEGFR-targeted therapies, we investigated the biological and potential therapeutic relevance of the HIF axis in ccRCC. Nuclear and cytoplasmic HIF1A and EPAS1/HIF2A expression were assessed by immunohistochemistry in tumours from 40 patients and correlated with clinicopathological parameters and cancer-specific survival. The functional effects of HIF pathway inhibitors (GN44028, KC7F2, and FM19G11) and sunitinib were analysed in VHL-mutant 786-O and VHL-wild-type Caki-1 cell lines using SRB viability assay, cell cycle analysis, wound closure assay, and RT-qPCR of HIF-related genes, with comparison to non-malignant HK-2 cells. TCGA-ccRCC data from advanced-stage patients (III–IV, *n* = 185) were analysed as a complementary transcriptomic context. Nuclear, but not cytoplasmic, HIF1A and EPAS1/HIF2A expression was associated with advanced stage and shorter survival in univariable analyses. GN44028 showed the most pronounced antiproliferative effect under tested conditions and was associated with broad suppression of HIF-related transcription, whereas sunitinib was associated with increased expression of selected HIF-related genes. GN44028 did not demonstrate clear selectivity over non-malignant HK-2 cells. Overall, nuclear HIF activation is associated with aggressive ccRCC biology, and broader HIF pathway modulation warrants further experimental investigation; however, the clinical findings remain exploratory, and therapeutic selectivity and translational relevance are not yet established.

## 1. Introduction

Clear-cell renal cell carcinoma (ccRCC) is the most common histological subtype of renal cell carcinoma and originates from epithelial cells of the proximal convoluted tubule. According to GLOBOCAN data, there were approximately 447,000 new cases of renal cell carcinoma (all subtypes) worldwide in 2022 [1]. Despite advances in surgical and systemic therapies, ccRCC is associated with unfavourable clinical outcomes. The 5-year survival rate for patients presenting with lymph node metastases at diagnosis ranges from 13% to 43%, depending on the tumour stage, grade, and molecular characteristics [2].

The pathogenesis of ccRCC is complex and involves multiple genetic and epigenetic alterations, among which the inactivation of the von Hippel–Lindau (VHL) tumour suppressor gene is a key molecular event. Under physiological conditions, intact pVHL regulates the stability of hypoxia-inducible factors HIF1A (Hypoxia-Inducible Factor 1 Alpha) and EPAS1/HIF2A (Endothelial PAS Domain Protein 1/Hypoxia-Inducible Factor 2) by promoting their ubiquitination and proteasomal degradation under normoxic conditions. In contrast, hypoxic conditions prevent this process, allowing HIF1A and EPAS1/HIF2A to accumulate, translocate to the nucleus, and function as transcription factors. These proteins activate a broad transcriptional program that enables cellular adaptation to oxygen deprivation, including alterations in glucose metabolism, pH regulation, cell survival, and angiogenesis [3]. When pVHL function is lost, as is characteristic of the majority of ccRCC cases, HIF1A and EPAS1/HIF2A signalling becomes constitutively active, independent of tissue oxygenation. This aberrant activation leads to the persistent induction of pro-tumourigenic pathways, including enhanced angiogenesis and metabolic reprogramming. ccRCC is increasingly recognised as a metabolically rewired malignancy tightly linked to VHL–HIF-driven alterations in central carbon metabolism [4,5]. Accumulating evidence indicates that HIF1A and EPAS1/HIF2A may contribute to epithelial–mesenchymal transition (EMT) by inducing transcriptional changes that alter cell morphology and adhesion properties, thereby promoting the invasion and metastatic dissemination of ccRCC cells [3,6].

Current perioperative and systemic therapies for advanced ccRCC predominantly include tyrosine kinase inhibitors (TKIs), such as sunitinib and cabozantinib, which target VEGFR (Vascular Endothelial Growth Factor Receptor)-driven angiogenic signalling [7,8]. However, their long-term clinical efficacy is limited by the frequent development of drug resistance in patients. Resistance to sunitinib has been linked to adaptive molecular changes within tumour cells, including the upregulation of hypoxia-related pathways and alternative pro-survival signalling cascades [9]. Immune checkpoint inhibitors, such as pembrolizumab, which targets PD-1 (Programmed cell death protein 1), have significantly improved survival outcomes in selected patient populations; nevertheless, their use is associated with immune-related adverse effects, including inflammatory complications affecting the lungs and endocrine organs [10]. Currently, mTOR (Mechanistic target of rapamycin) kinase inhibitors (e.g., everolimus) are reserved for patients who are not eligible for PD-1- or VEGFR-targeted therapies. Despite these therapeutic advances, the overall treatment outcomes for advanced ccRCC remain suboptimal, underscoring the urgent need for novel molecular targets and therapeutic strategies [11]. In this context, the direct targeting of hypoxia-inducible factors has emerged as a promising therapeutic approach. Belzutifan, a selective EPAS1/HIF2A inhibitor, has been approved for the treatment of advanced renal cell carcinoma in selected clinical settings [12]. Belzutifan inhibits EPAS1/HIF2A-driven transcriptional activity, resulting in reduced tumour growth and delayed disease progression [13]. However, its clinical use is associated with adverse effects, such as anaemia, fatigue, and treatment-related hypoxia, highlighting the need for careful patient selection and further optimisation of HIF-targeted therapies [14].

In our previous studies, we demonstrated that increased expression of HIF1A and EPAS1/HIF2A, as well as elevated levels of their downstream target VEGF, were associated with shorter overall survival in patients with ccRCC [15,16]. Given the recent clinical introduction of HIF2A-targeted therapy and the limited understanding of the distinct and overlapping roles of HIF1A and EPAS1/HIF2A in renal cancer biology, these observations prompted us to systematically investigate the pharmacological modulation of the HIF axis in renal cell carcinoma models. In this context, we included GN44028, an investigational compound reported to modulate HIF1A-related pathways, along with reference HIF pathway inhibitors and standard therapy.

The present study evaluates the effects of selected HIF pathway-modulating compounds on viability, cell-cycle distribution, wound closure dynamics, and HIF-related transcription in ccRCC cell lines, with the aim of identifying biologically relevant HIF-dependent effects across integrated experimental systems.

## 2. Results

### 2.1. Clinicopathological Characteristics of the Patients

Forty patients with histologically confirmed clear-cell renal cell carcinoma were included in the analysis. The clinicopathological characteristics of the analysed patient cohort are presented in Table 1. The cohort comprised predominantly male patients (*n* = 29), with 11 female cases. The average age was 67.35 ± 9.52 years (range, 46–86 years). Tumour extent was classified according to the UICC TNM 8th edition criteria [17]. The majority of cases represented advanced disease (stage III–IV, *n* = 29), whereas 11 tumours were confined to stage I–II disease. Grading based on the Fuhrman/WHO/ISUP system [18] showed representation across the full spectrum of differentiation, from grade 1 (*n* = 3) to grade 4 (*n* = 12). Patients were monitored in an oncological outpatient setting with a mean follow-up duration of 35 months (range, 6–120 months). All deaths (*n* = 20) were associated with ccRCC progression, based on documentation from the oncology clinic, and the median cancer-specific survival (CSS) was 30 months.

### 2.2. Nuclear Immunoexpression of HIF1A and EPAS1/HIF2A Proteins

**Figure 1:**
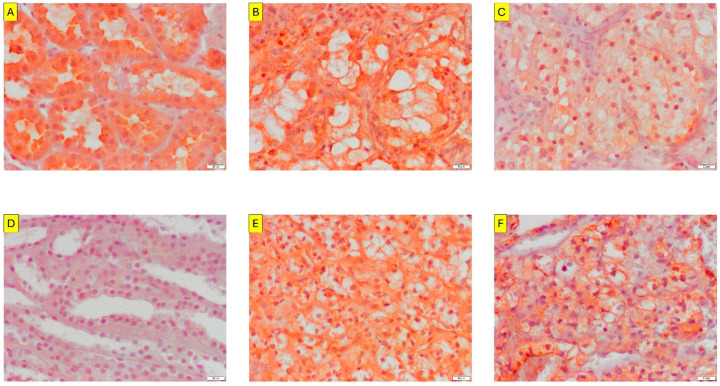
Representative immunohistochemical (IHC) staining patterns of HIF1A and EPAS1/HIF2A in normal kidney tissue and clear-cell renal cell carcinoma (ccRCC). (**A**) shows strong cytoplasmic HIF1A immunoreactivity in epithelial cells of the proximal renal tubules in normal kidney tissue, with no nuclear staining or reactivity in stromal cells. (**B**) shows strong cytoplasmic and focal nuclear HIF1A expression in ccRCC tumour cells. (**C**) illustrates weak nuclear and moderate cytoplasmic HIF1A staining in ccRCC, with the absence of immunoreactivity in stromal cells. (**D**) shows the normal kidney medulla, including the loops of Henle and collecting ducts, showing the absence of HIF2A immunoreactivity. (**E**) shows strong cytoplasmic EPAS1/HIF2A expression in ccRCC tumour cells with minimal nuclear staining. (**F**) demonstrates moderate combined nuclear and cytoplasmic HIF2A expression in ccRCC; residual preserved tubules of the thin limb of the loop of Henle are visible and remain immunonegative, corresponding to the structures shown in (**D**). Stromal cells are consistently negative. Images were acquired using an Olympus CX31 microscope equipped with SC50 camera and the CellSens software (ver. 4.1.1, Olympus, Hamburg, Germany). Original magnification, ×200. Scale bar in bottom right corner = 20 µm.

Immunohistochemical (IHC) analysis revealed distinct localisation patterns of HIF1A and EPAS1/HIF2A proteins in normal kidney tissue compared with that in ccRCC (Figure 1). In non-neoplastic kidney samples, strong cytoplasmic HIF1A immunoreactivity was observed in the epithelial cells of the proximal renal tubules, whereas nuclear staining was absent (Figure 1A). In contrast, tubular structures of the renal medulla, including the loops of Henle and collecting ducts, showed no detectable HIF1A or EPAS1/HIF2A expression (Figure 1D), serving as an internal negative control. Stromal cells were consistently immunonegative in all the normal kidney samples.

In ccRCC tissues, both HIF1A and EPAS1/HIF2A exhibited heterogeneous but tumour-restricted expression patterns. Tumour cells frequently exhibited strong cytoplasmic staining, accompanied by variable nuclear immunoreactivity (Figure 1B,C,E,F). Importantly, the nuclear accumulation of HIF1A and EPAS1/HIF2A was observed exclusively in neoplastic cells and was absent in the surrounding stromal components and residual non-neoplastic tubular structures (Figure 1C,F). In the selected tumour areas, remnants of preserved thin limbs of the loop of Henle remained immunonegative for HIF proteins (Figure 1F), further underscoring the specificity of HIF nuclear localisation in malignant cells.

These findings indicate that while cytoplasmic HIF expression is detectable in both normal and malignant renal epithelial cells, the nuclear localisation of HIF1A and EPAS1/HIF2A represents a tumour-specific feature of ccRCC and reflects their transcriptionally active state. Based on this spatial restriction and biological relevance, subsequent quantitative and prognostic analyses focused on nuclear HIF immunoreactivity.

**Figure 2:**
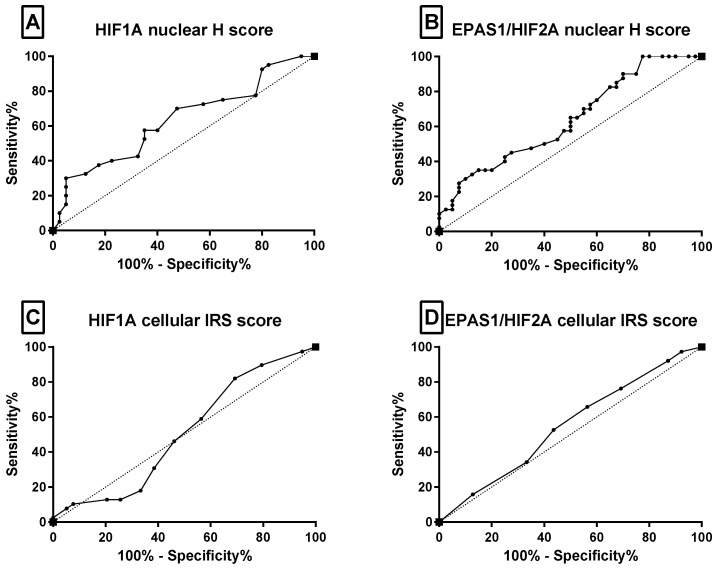
Receiver operating characteristic (ROC) curves evaluating the prognostic performance of HIF1A and EPAS1/HIF2A immunoexpression for cancer-specific survival in patients with clear-cell renal cell carcinoma (ccRCC) (*n* = 40). (**A**) HIF1A nuclear H-score (AUC = 0.635, 95% CI 0.513–0.757, *p* = 0.038), (**B**) EPAS1/HIF2A nuclear H-score (AUC = 0.638, 95% CI 0.518–0.759, *p* = 0.033), (**C**) HIF1A cytoplasmic IRS score (AUC = 0.507, 95% CI 0.376–0.638, *p* = 0.913), (**D**) EPAS1/HIF2A cytoplasmic IRS score (AUC = 0.549, 95% CI 0.419–0.678, *p* = 0.463). The dashed black diagonal line represents the reference line of no discrimination (AUC = 0.5).

**Figure 3:**
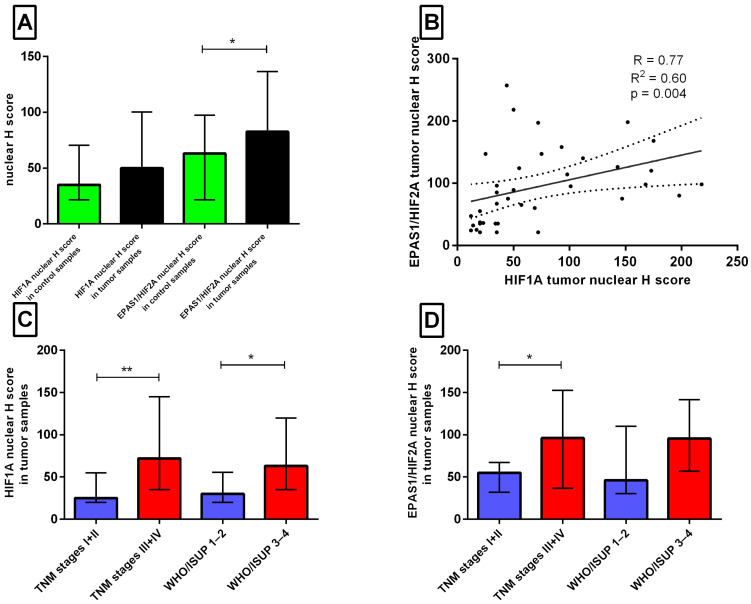
Nuclear expression of HIF1A and EPAS1/HIF2A in clear-cell renal cell carcinoma (ccRCC) and adjacent non-tumour kidney tissue. (**A**) Nuclear H-scores of HIF1A and HIF2A in paired non-tumour vs. tumour tissues (*n* = 40; * *p* < 0.05, Mann–Whitney U test). (**B**) Spearman correlation between nuclear HIF1A and EPAS1/HIF2A H-scores in tumour samples (R = 0.77, R^2^ = 0.60, *p* = 0.004). (**C**) Nuclear HIF1A H-score in tumour samples stratified by TNM stage (I + II vs. III + IV) and WHO/ISUP histological grade (1–2 vs. 3–4). (**D**) Nuclear EPAS1/HIF2A H-score in tumour samples stratified by TNM stage (I + II vs. III + IV) and WHO/ISUP histological grade (1–2 vs. 3–4). Box-and-whisker plots (**A**,**C**,**D**) show the median, interquartile range, and 10–90th percentiles. * *p* < 0.05, ** *p* < 0.01, Mann–Whitney U test. Solid line indicates the linear regression line; dotted lines represent the 95% confidence bands in (**B**).

To identify an analytically useful scoring approach for exploratory outcome analysis, receiver operating characteristic (ROC) analysis was performed (Table 2 and Figure 2). ROC-based thresholding has been used in previous renal cancer studies to define biologically informed expression categories for HIF-related markers [19]. Cytoplasmic IRS scores for both HIF1A and EPAS1/HIF2A showed no discriminatory power (AUC = 0.507 and 0.549, respectively; *p* > 0.46). In contrast, the nuclear H-score showed modest but statistically significant discriminatory performance: HIF1A AUC = 0.635 (95% CI: 0.513–0.757, *p* = 0.038) and EPAS1/HIF2A AUC = 0.638 (95% CI: 0.518–0.759, *p* = 0.033). The optimal cut-offs determined by Youden’s index were HIF1A ≥ 32 (sensitivity 70%, specificity 60%) and EPAS1/HIF2A ≥ 62.5 (sensitivity 75%, specificity 55%). Therefore, the nuclear H-score was used for subsequent exploratory analyses. Using these cut-offs, elevated nuclear HIF1A (≥32) was detected in 28/40 (70%) and elevated nuclear EPAS1/HIF2A (≥62.5) in 25/40 (63%) tumour samples. Nuclear HIF1A expression was significantly associated with advanced disease (*p* = 0.007; Table 3). Quantitative comparison of H-scores revealed a 1.5-fold higher nuclear EPAS1/HIF2A expression in tumours than in adjacent non-tumour tissues (*p* = 0.0296, Figure 3A). Within the tumour samples, nuclear HIF1A and EPAS1/HIF2A H-scores were positively correlated (Spearman’s rho = 0.77, R^2^ = 0.60, *p* = 0.004; Figure 3B). Advanced TNM stage was associated with 2.6-fold higher nuclear HIF1A and 2.0-fold higher nuclear EPAS1/HIF2A expression (Figure 3C,D), and a higher ISUP grade was associated with a 2.0-fold increase in nuclear HIF1A (*p* < 0.05). Overall, these findings suggest that nuclear HIF1A and EPAS1/HIF2A expression is associated with adverse clinicopathological features in this cohort, whereas cytoplasmic expression did not show discriminatory value.

Patients with ccRCC (*n* = 40) were stratified according to clinicopathological parameters, including age, sex, tumour size, laterality, ISUP histological grade, and TNM stage. Nuclear expression of HIF1A and EPAS1/HIF2A was evaluated using the H-score method with ROC-derived cut-offs (HIF1A ≥ 32, EPAS1/HIF2A ≥ 62.5) and dichotomised into low (↓) and high (↑) expression groups. Associations between protein expression and clinical variables were assessed using the two-sided Fisher’s exact test, and the results are summarised in Table 3. Nuclear HIF1A expression (↑ in 28/40, 70%) was significantly enriched in advanced tumours (TNM stage III + IV: 24/28 vs. 4/12 in stage I + II; *p* = 0.007) and showed a strong trend toward higher ISUP grade (3 + 4:21/28 vs. 5/12 in 1 + 2; *p* = 0.070). Nuclear EPAS1/HIF2A expression (↑ in 25/40, 63%) demonstrated a borderline association with advanced stage (III + IV: 21/25 vs. 4/15; *p* = 0.065) and grade (*p* = 0.089) of the disease. No significant correlations were observed between age (<70 vs. ≥70 years), sex, tumour size (≤7 vs. >7 cm), or laterality (left vs. right). These findings indicate that nuclear accumulation of HIF1A, and to a lesser extent, EPAS1/HIF2A, is associated with aggressive tumour behaviour in ccRCC, particularly in advanced-stage disease.

### 2.3. Nuclear Immunoexpression of HIF Proteins and Patient Outcomes

**Figure 4:**
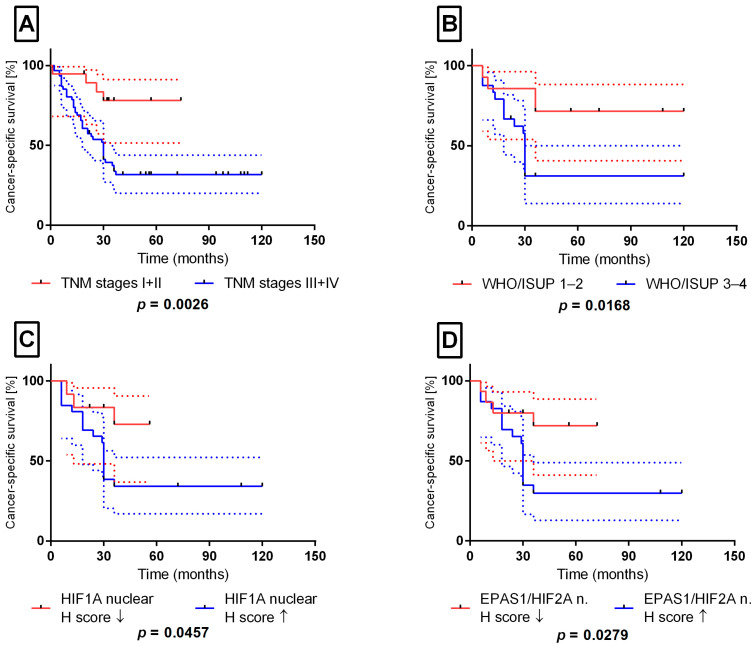
Survival analysis of the ccRCC cohort. Cancer-specific survival (CSS) was evaluated using Kaplan–Meier methodology in 40 patients with clear-cell renal cell carcinoma (20 cancer-related events). Stratification was performed according to the tumour TNM stage (**A**), WHO/ISUP histological grade (**B**), nuclear H-score of HIF1A (**C**), and nuclear H-score of EPAS1/HIF2A (**D**). Expression thresholds were established using ROC curve analysis with Youden’s index. Solid lines represent Kaplan–Meier survival curves, while dashed lines indicate the 95% confidence intervals. Arrows (↓ and ↑) denote values below and above the predefined cut-off thresholds, respectively (low vs. high expression groups). Statistical significance was assessed using the log-rank test. *n*—nuclear localisation.

Advanced TNM stage (Figure 4A) and higher WHO/ISUP grade (Figure 4B) were associated with shorter cancer-specific survival (CSS). Higher nuclear immunoreactivity of HIF1A and EPAS1/HIF2A was also associated with shorter CSS in the univariable analysis (Figure 4C,D). Cox proportional hazards analysis is summarised in Table 4. Because the number of events was limited (*n* = 20) and the full multivariable model was unstable, LASSO-penalised Cox regression was used to derive a more parsimonious exploratory model with fewer variables. This model retained tumour stage (III + IV vs. I + II), ISUP grade (3 + 4 vs. 1 + 2), and nuclear HIF1A expression (high vs. low) as significant variables. Bootstrap resampling (B = 10,000) supported the stability of the stage and grade estimates, whereas the effect of nuclear HIF1A remained non-significant after adjustment for confounding factors. Accordingly, the clinical analyses should be interpreted as exploratory. Although nuclear HIF1A and EPAS1/HIF2A were associated with survival in univariable analyses, established clinicopathological parameters remained the dominant prognostic factors in this dataset.

### 2.4. Effects of Selected Inhibitors on the Viability of 786-O, Caki-1, and HK-2 Cells

**Figure 5:**
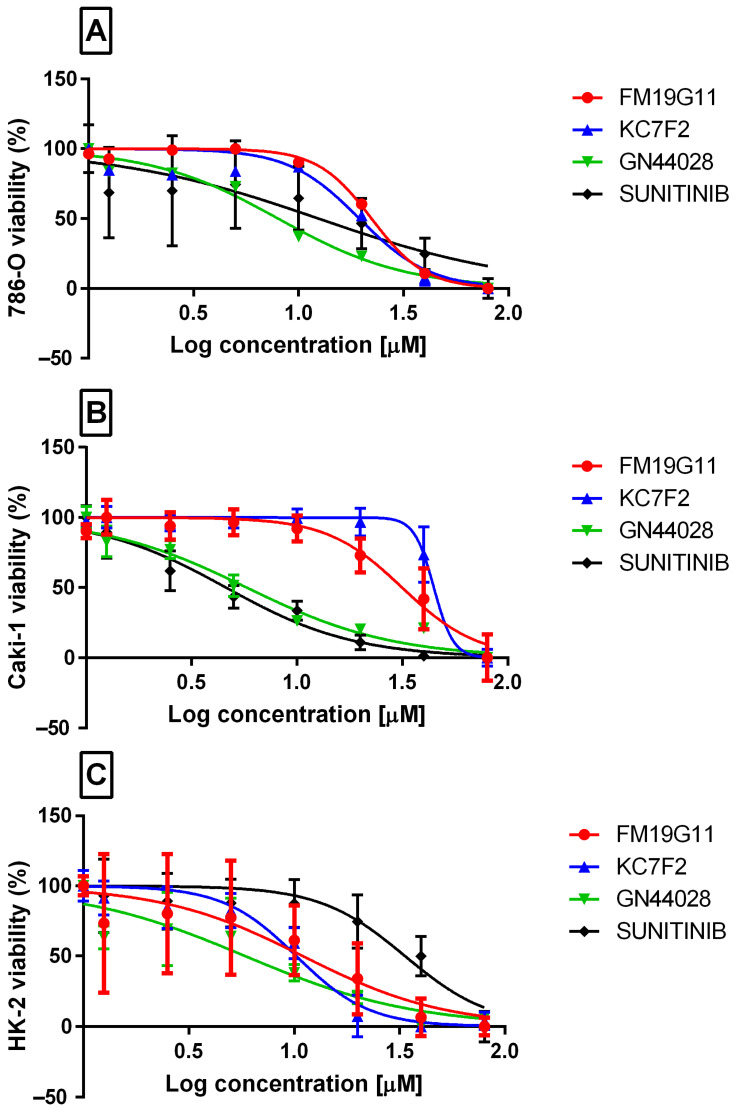
Cytotoxicity of HIF inhibitors and sunitinib in renal cell lines: (**A**) 786-O, (**B**) Caki-1, and (**C**) HK-2. Dose–response curves (SRB, 72 h of incubation). Data are presented as mean ± SD from independent biological experiments, each measured in technical replicates (*n* = 6). Curves were fitted using four-parameter logistic regression.

The sulforhodamine B (SRB) assay showed dose-dependent reductions in viability in both ccRCC cell lines (786-O and Caki-1) and the non-malignant proximal tubule cell line HK-2 following treatment with GN44028, KC7F2, FM19G11, and sunitinib (Figure 5A–C). Exposure times of 24, 48, and 72 h were initially tested; the 72 h time point was selected for final IC_50_ calculations because it yielded the most informative dose–response curves. The half-maximal inhibitory concentrations (IC_50_, 72 h) from six independent experiments are presented in Table 5. GN44028 showed the lowest IC_50_ values across the tested cell lines: 3.39 ± 0.51 µM in HK-2, 7.99 ± 1.03 µM in 786-O, and 5.77 ± 0.74 µM in Caki-1 cells. Sunitinib showed greater activity in Caki-1 cells (IC_50_ = 4.61 ± 0.59 µM) than in 786-O cells (IC_50_ = 12.47 ± 1.61 µM) and HK-2 cells (IC_50_ = 33.53 ± 4.12 µM). FM19G11 and KC7F2 exhibited intermediate potency (IC_50_ range 10.23–44.61 µM), with Caki-1 cells being the least sensitive to both compounds. Overall, GN44028 was the most potent compound in this assay; however, because the lowest IC_50_ value was observed in non-malignant HK-2 cells, these data do not support preferential cytotoxicity toward malignant cells under the tested conditions.

### 2.5. Effect of Inhibitors on the Cell Cycle

**Figure 6:**
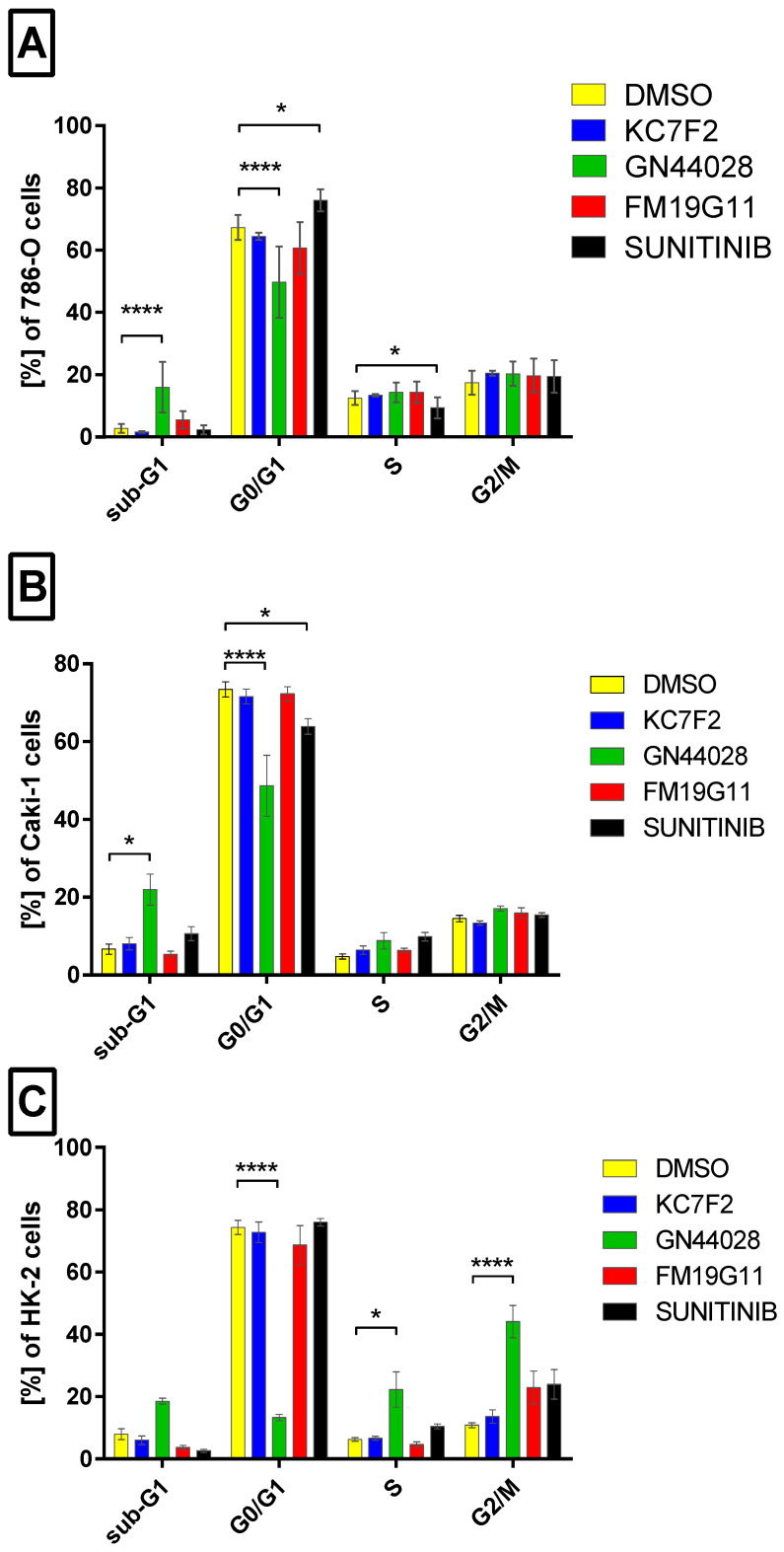
Cell cycle analysis by propidium iodide staining and flow cytometry in 786-O (**A**), Caki-1 (**B**), and HK-2 (**C**) cells treated with KC7F2, FM19G11, GN44028, and sunitinib at IC_50_ concentrations for 24 h. Bar graphs show mean ± SD from independent biological experiments, each measured in technical replicates (*n* = 3–19). GN44028 was associated with an increased sub-G1 fraction in ccRCC cell lines and G2/M arrest in HK-2 cells. Sunitinib induces G0/G1 arrest in 786-O cells. **** *p* < 0.0001, * *p* < 0.05 vs. DMSO (ANOVA with Dunnett’s test).

Flow cytometry analysis of cell cycle distribution revealed that only GN44028 and sunitinib significantly altered the cell cycle profiles at their respective IC_50_ concentrations (Figure 6). In 786-O and Caki-1 ccRCC cell lines, GN44028 induced a marked increase in the sub-G1 fraction (16.8 ± 6.7% and 23.8 ± 10.3%, respectively, vs. 2.7 ± 1.2% and 6.7 ± 3.3% in DMSO; *p* < 0.001), accompanied by a significant reduction in the G0/G1 phase (to 50.2 ± 11.1% and 49.1 ± 21.5%), consistent with apoptosis induction (Figure 6A,B). Sunitinib caused G0/G1 arrest in 786-O cells (76.2 ± 3.5% vs. 67.6 ± 4.5% in the control; *p* < 0.01) with no significant sub-G1 increase (Figure 6A). In the HK-2 normal renal cell line, GN44028 induced a dose-dependent G2/M arrest (44.1 ± 13.0% vs. 10.8 ± 2.6% in DMSO; *p* < 0.001) and a sharp decrease in G0/G1 (13.6 ± 2.5%), with no elevation in sub-G1, suggesting cytostatic rather than cytotoxic effects (Figure 6C). KC7F2 and FM19G11 had no significant impact on cell cycle distribution in any tested cell line.

### 2.6. Effects of HIF1A/EPAS1/HIF2A Inhibitors on Wound Closure Dynamics in 786-O and Caki-1 Cells

**Figure 7:**
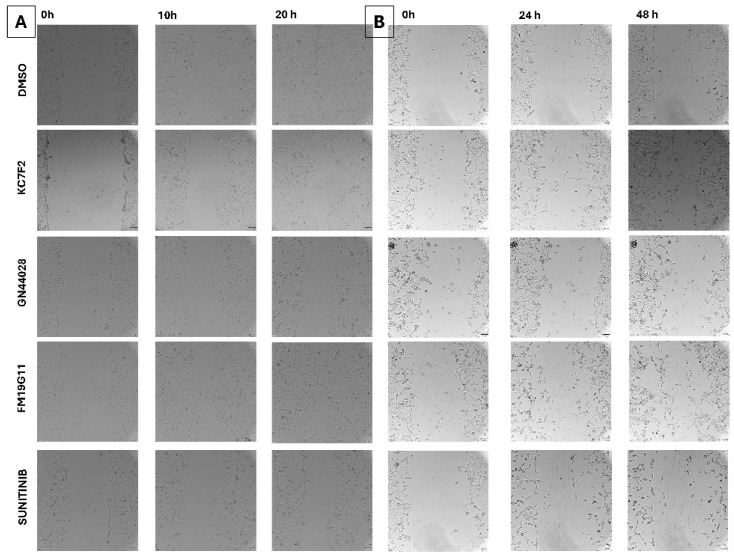
Representative images from the wound closure assay. (**A**) 786-O cells were imaged at 0, 10, and 20 h. (**B**) Caki-1 cells were imaged at 0, 24, and 48 h. The compounds are arranged from top to bottom: DMSO, KC7F2, GN44028, FM19G11, and sunitinib. Scale bar: 100 µm. Magnification 400×.

**Figure 8:**
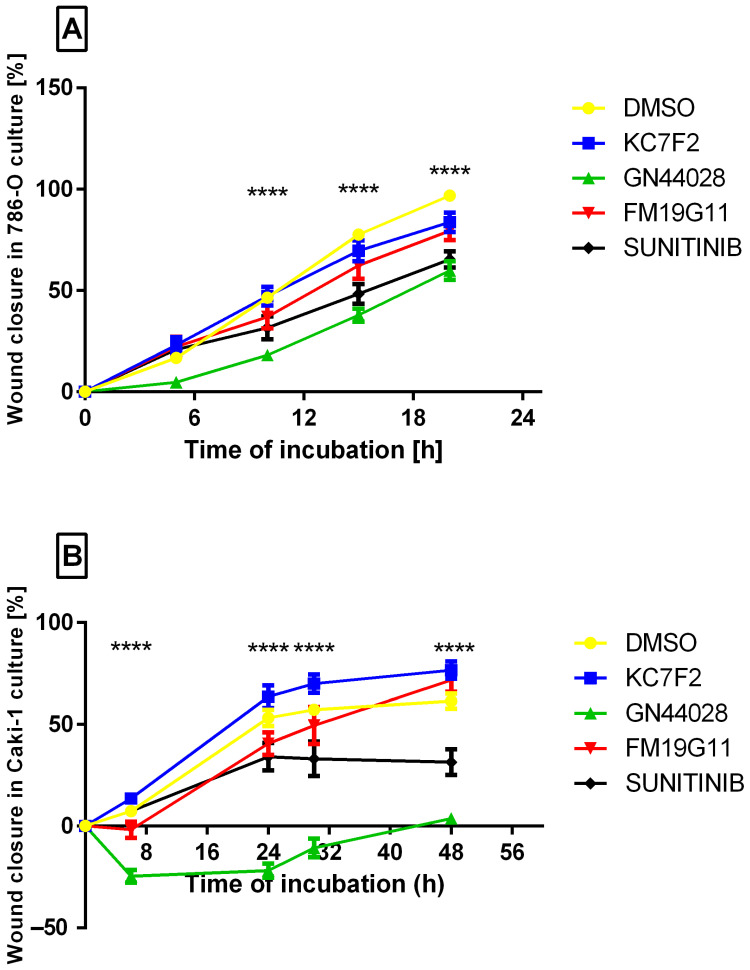
Quantitative analysis of wound closure dynamics. (**A**) 786-O cells (0–20 h). (**B**) Caki-1 cells (0–48 h). Y-axis: Wound closure in culture [%]. The compounds are arranged from top to bottom: DMSO (yellow), KC7F2 (blue), GN44028 (green), FM19G11 (red), and sunitinib (black). Data are presented as mean ± SD from independent biological experiments, each measured in technical replicates (*n* = 18–23). **** *p* < 0.0001 vs. DMSO (one-way ANOVA + Dunnett’s test).

A wound closure assay was performed on Caki-1 and 786-O cell lines. HK-2 cells did not exhibit wound healing under control conditions (even after 48 h) and were therefore not included in this analysis. Images were recorded at 5–6 h intervals for 786-O and Caki-1 cells separately (Figure 7A,B), while the plots are presented in Figure 8A,B. The experiment duration was set to 20 h for 786-O cells and 48 h for Caki-1 cells, which corresponded to complete gap closure in the control cultures. The observed effects should be interpreted in the context of concurrent antiproliferative and cell cycle effects and therefore do not represent a direct measure of migratory capacity. GN44028 and sunitinib were associated with reduced wound closure in 786-O cells (61.4 ± 8.09% and 67.7 ± 12.3% wound closure, respectively, versus control; Figure 7A and Figure 8A). In Caki-1 cells, GN44028 was associated with markedly reduced wound closure (4.9 ± 4.8% at 48 h vs. control; Figure 7B and Figure 8B), whereas sunitinib produced a slower but still detectable reduction in wound closure dynamics. However, as wound closure under the tested conditions was likely influenced by concurrent antiproliferative and cell cycle effects, these findings should not be interpreted as direct evidence of reduced migratory capacity. In contrast, FM19G11 and KC7F2 were associated with faster wound closure in Caki-1 cells (*p* < 0.05) (Figure 7B and Figure 8B).

### 2.7. qPCR Results for HIF-Regulated Genes

**Figure 9:**
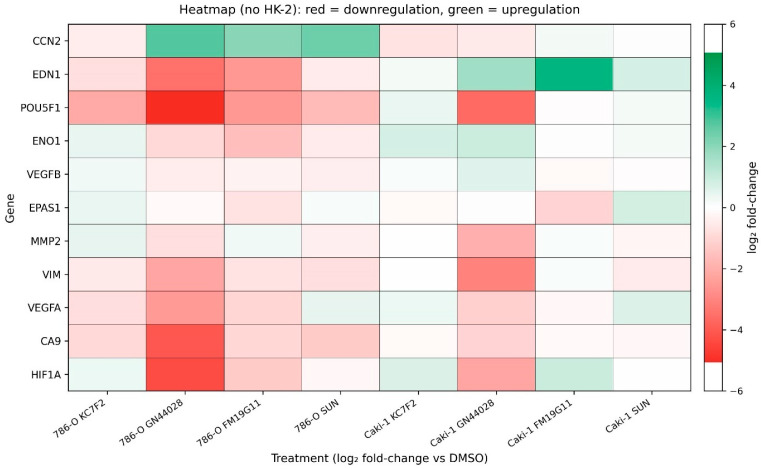
Heatmap of transcriptional responses of hypoxia- and HIF-regulated genes in renal cell carcinoma cell lines following pharmacological inhibition of the HIF pathway. The heatmap shows log_2_ (fold change) values of mRNA expression relative to vehicle-treated controls (DMSO) for 786-O and Caki-1 cells after 72 h of treatment with KC7F2, GN44028, FM19G11, or sunitinib (SUN) at IC_50_ concentrations. Normal proximal tubule cells (HK-2) were excluded from visualisation. All qPCR data are presented as log_2_ fold change relative to the vehicle-treated controls (DMSO). Red, green, and white colours indicate downregulation, upregulation, and no change in expression, respectively. The colour scale represents log_2_FC values ranging from −5 to +5. Genes were hierarchically clustered (average linkage, Euclidean distance), and treatment conditions were ordered manually. Each value represents the mean of two independent biological experiments, each with three technical replicates.

qPCR analyses of selected hypoxia- and HIF-related genes suggested compound- and cell line-specific transcriptional responses (Figure 9). In 786-O cells, GN44028 induced broad suppression of the HIF-driven transcriptional program, including downregulation of *HIF1A* mRNA by 94% (log_2_FC = −4.08, *p* < 0.0001), *POU5F1* by 96% (log_2_FC = −4.64, *p* < 0.0001), *VIM* by 83% (log_2_FC = −2.56, *p* < 0.0001), and *VEGFA* by 81% (log_2_FC = −2.40, *p* < 0.0001), as well as significant repression of *EPAS1/HIF2A* (log_2_FC = −0.69). This broad inhibitory profile was accompanied by a marked upregulation of *CCN2* (6.1-fold, log_2_FC = +2.61, *p* < 0.0001; Kruskal–Wallis test with Dunn’s post-test). In the more resistant Caki-1 cell line, GN44028 retained strong suppressive activity against *VIM* (89%, log_2_FC = −3.47, *p* < 0.0001), *POU5F1* (93%, log_2_FC = −3.84, *p* < 0.0001), *HIF1A* (79%, log_2_FC = −2.25, *p* < 0.0001), *VEGFA* (53%, log_2_FC = −1.10, *p* < 0.001), and *EPAS1/HIF2A* (log_2_FC = −1.06). In contrast, sunitinib treatment produced a distinct transcriptional response. In 786-O cells, sunitinib was associated with increased expression of *EPAS1/HIF2A* (log_2_FC = +0.13), *VEGFA* (1.6-fold, log_2_FC = +0.69, *p* < 0.05), and *EDN1* (log_2_FC = +0.75), while failing to suppress the core HIF1A target genes. In Caki-1 cells, sunitinib increased *EPAS1/HIF2A* expression (log_2_FC = +0.81) and showed no consistent inhibitory effect on *VEGFA* or *EDN1*. The reference *HIF1A* translation inhibitors KC7F2 and FM19G11 displayed intermediate and gene-selective effects, lacking the consistent multi-gene suppression observed with GN44028. Overall, GN44028 showed the most consistent pattern of downregulation across the examined genes, whereas sunitinib was associated with treatment-related upregulation of selected HIF-related transcripts. These patterns were directionally consistent across biological repeats but require confirmation in experiments with expanded biological replication. Given that these data are based on two independent biological replicates, they should be interpreted as exploratory and hypothesis-generating only.

### 2.8. TCGA-Based Transcriptomic Context of HIF Pathway Genes in Advanced ccRCC

**Figure 10:**
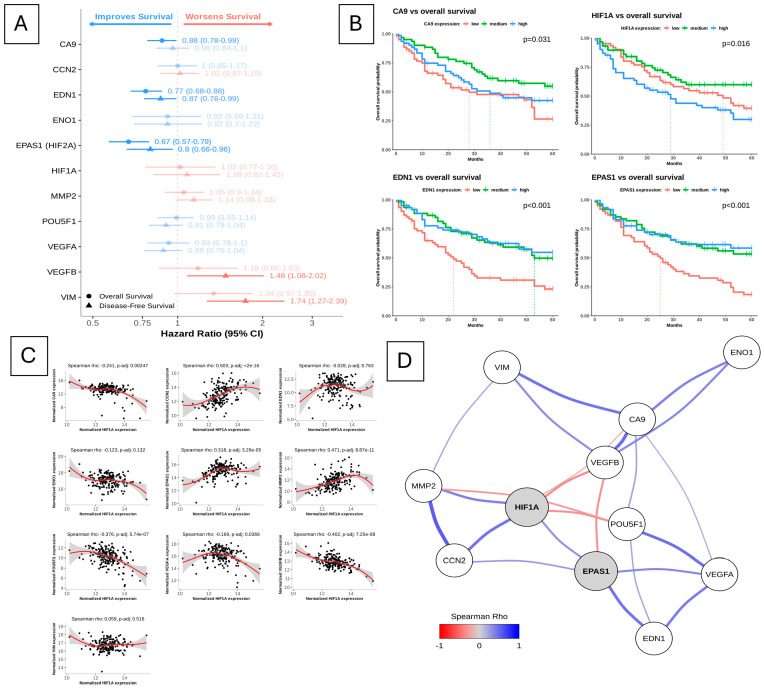
TCGA-based analysis of HIF pathway gene expression and clinical relevance in advanced clear-cell renal cell carcinoma (ccRCC). (**A**) Forest plot showing hazard ratios (HR) with 95% confidence intervals for overall survival (OS) in TCGA-ccRCC patients with advanced disease (pathological stages III–IV, *n* = 185). Patients were stratified into terciles according to VST-normalized gene expression levels. HR < 1 indicates improved survival associated with higher gene expression, whereas HR > 1 indicates increased risk. (**B**) Kaplan–Meier survival curves for selected HIF pathway-related genes stratified by expression terciles (low, medium, high). Overall survival (OS) is shown for *CA9*, *HIF1A*, *EDN1*, and *EPAS1/HIF2A* (**B**). *p*-values were calculated using the log-rank (Mantel–Cox) test. (**C**) Spearman correlation analysis between HIF1A expression and selected hypoxia- and HIF-regulated genes in advanced TCGA-ccRCC samples. Scatter plots show VST-normalized expression values with locally estimated scatterplot smoothing (LOESS) curves. Corresponding Spearman’s rho coefficients and Benjamini–Hochberg-adjusted *p*-values are indicated. (**D**) Correlation network illustrating molecular interactions among HIF pathway-associated genes in advanced ccRCC. Nodes represent genes, and edges indicate significant Spearman correlations (adjusted *p* < 0.05). Edge colour denotes direction and strength of correlation (blue, positive; red, negative), and edge width reflects correlation magnitude.

To provide a complementary transcriptomic context for the experimental findings, we analysed transcriptomic and survival data from the TCGA-ccRCC cohort, restricted to patients with advanced disease (pathological stages III–IV, *n* = 185). Forest plot analysis revealed gene-specific, directionally distinct associations with patient outcomes (Figure 10A). Cox proportional hazards analysis showed that higher expression of *HIF1A*, *VIM, MMP2*, and *VEGFB* was associated with an increased risk of death, whereas *EPAS1/HIF2A*, *CA9*, and *EDN1* showed an inverse relationship, with lower expression correlating with poorer overall survival. Kaplan–Meier survival analysis showed consistent trends (Figure 10B), with low expression of *EDN1*, *CA9*, and *EPAS1/HIF2A* associated with unfavourable OS, whereas higher *HIF1A* expression identified a subgroup of patients with reduced survival probability. Reduced *EPAS1/HIF2A* expression was associated with a more aggressive disease course (*p* < 0.001), highlighting the context-dependent associations of EPAS1/HIF2A signalling in advanced ccRCC.

Correlation analysis demonstrated that *HIF1A* expression was embedded within a tightly interconnected transcriptional network, showing significant associations with *EPAS1/HIF2A*, *VEGFA*, *EDN1*, *CCN2*, *VIM*, and *MMP2* (Figure 10C). Notably, *HIF1A* correlated positively with mesenchymal and extracellular matrix-related genes, while displaying inverse associations with selected angiogenic regulators, such as *VEGFB*, supporting its role as a central transcriptional regulator associated with aggressive tumour phenotypes (Figure 10D). Visualisation of the correlation network further highlighted HIF1A and *EPAS1/HIF2A* as central, but functionally distinct, nodes within the hypoxia-associated transcriptional landscape (Figure 10D).

Overall, the TCGA analysis identified gene-expression patterns broadly consistent with HIF-associated biological heterogeneity in advanced ccRCC. These data provide a complementary transcript-level context for the experimental findings, but they should not be interpreted as direct validation of the tissue-based IHC results or of the clinical cohort.

## 3. Discussion

Clear-cell renal cell carcinoma (ccRCC) is a paradigmatic example of a malignancy driven by dysregulation of the VHL–HIF signalling axis. Loss of functional VHL protein leads to constitutive stabilization of hypoxia-inducible factors, particularly HIF1A and EPAS1/HIF2A, resulting in persistent activation of hypoxia-responsive transcriptional programs even under normoxic conditions [3]. These pathways regulate angiogenesis, metabolic reprogramming, epithelial–mesenchymal transition (EMT), and invasive behaviour, thereby contributing to the aggressive phenotype and therapeutic resistance characteristics of advanced ccRCC [3,6,12]. Our group has been investigating components of this pathway for several years, both at the tissue and cellular level, and the present study integrates clinical, experimental, and in silico data to refine the biological and therapeutic relevance of HIF1A and EPAS1/HIF2A in ccRCC [9,15,16].

An important limitation of the clinical component is the relatively small cohort size (*n* = 40) with a limited number of events, which increases the risk of overfitting in multivariable analyses. To address this, we employed penalised regression (LASSO) and bootstrap resampling; nevertheless, these approaches confirmed that classical clinicopathological parameters, particularly tumour stage and grade, remain the primary drivers of prognosis in this dataset. Consequently, the observed associations involving HIF-related markers should be considered exploratory and hypothesis-generating, rather than definitive evidence of independent prognostic value. A key observation of this study was the differential subcellular distribution of HIF1A and EPAS1/HIF2A in normal kidney tissue versus ccRCC. In the non-neoplastic renal parenchyma, HIF1A expression was restricted to the cytoplasm of proximal tubular epithelial cells, whereas medullary structures, including the loops of Henle and collecting ducts, were immunonegative. This physiological pattern contrasts sharply with that observed in ccRCC, where both HIF1A and EPAS1/HIF2A exhibit prominent nuclear accumulation selectively within tumour cells. Similar observations have been reported in independent IHC-based studies, where nuclear localisation of HIF proteins, rather than cytoplasmic expression, was shown to correlate more strongly with aggressive clinicopathological features and adverse survival outcomes in renal and other solid tumours than cytoplasmic expression. In ccRCC, nuclear HIF1A and EPAS1/HIF2A expression have been proposed as surrogate markers of transcriptionally active hypoxia signalling, whereas cytoplasmic staining alone lacks prognostic significance [20,21]. The biological relevance of nuclear HIF localisation has been reported in independent clinical cohorts. Sitaram et al. demonstrated that nuclear expression of HIF1A and HIF3A, but not cytoplasmic expression, was associated with adverse clinicopathological features and reduced CSS in ccRCC, whereas cytoplasmic expression lacked prognostic significance [20]. Similarly, in colorectal cancer, Cao et al. showed that nuclear HIF1A expression correlated with tumour stage, metastatic spread, and poor overall survival, whereas cytoplasmic localisation was not independently prognostic [21]. Together with our data, these findings support the concept that nuclear accumulation of HIF proteins reflects their transcriptionally active state and represents a biologically and clinically meaningful readout of HIF pathway activation across solid tumours [20,21].

Importantly, nuclear HIF (HIF1A and EPAS1/HIF2A) immunoreactivity was absent in stromal cells and residual non-neoplastic tubular elements embedded within the tumour tissue, indicating that nuclear localisation is not merely a reflection of the hypoxic microenvironment but rather a tumour-specific regulatory feature. To our knowledge, such strict spatial restriction of nuclear HIF localisation exclusively to malignant epithelial cells, with complete absence in stromal and residual non-neoplastic tubular compartments, has not been systematically documented in previous studies on ccRCC. This spatial restriction supports the concept that nuclear HIF activity, rather than overall protein abundance, is the biologically relevant determinant of transcriptional reprogramming in ccRCC. These findings are consistent with the canonical model of VHL-deficient ccRCC, in which the stabilisation and nuclear translocation of HIF proteins drive the sustained activation of hypoxia-responsive gene networks [22]. Our data extend this model by demonstrating that cytoplasmic HIF (HIF1A and EPAS1/HIF2A) expression alone lacks prognostic relevance, whereas nuclear accumulation correlates with aggressive clinicopathological features and poor outcomes. This observation aligns with previous reports suggesting that the subcellular localisation of HIF1A and EPAS1/HIF2A proteins may outperform total expression levels as a biomarker of functional HIF signalling [20].

Using ROC-guided cut-offs, we demonstrated that high nuclear HIF1A expression is significantly enriched in advanced tumours and correlates with advanced TNM stage and higher ISUP grade. The use of ROC-based approaches to define clinically meaningful cut-offs for HIF1A and EPAS1/HIF2A expression is supported by recent imaging–pathology correlation studies, which demonstrate that the quantitative assessment of EPAS1/HIF2A expression can reflect biological activity and may be associated with therapeutic response in ccRCC. While ROC-derived cut-offs do not imply immediate clinical applicability, they provide a biologically informed framework for dichotomising HIF activity states and reducing the arbitrariness inherent to semi-quantitative IHC scoring. These findings support the validity of ROC-guided IHC scoring as a biologically informed rather than an arbitrary method of risk stratification [19]. However, it is important to acknowledge that the discriminative performance of the ROC-derived thresholds for nuclear HIF1A and EPAS1/HIF2A was only moderate. The observed AUC values (~0.63) do not support clinical diagnostic applications and should be interpreted with caution. Notably, nuclear HIF assessment demonstrated better performance than the overall tissue-level expression, but the difference was not statistically significant. This observation reinforces the concept that subcellular localisation, reflecting the transcriptionally active form of HIF proteins, may be more biologically informative than total protein or mRNA abundance. Therefore, although nuclear HIF immunoreactivity cannot be considered a standalone diagnostic or prognostic biomarker, it may still provide insight into the functional activation of hypoxia-driven pathways. Although both HIF1A and EPAS1/HIF2A nuclear expression were associated with shorter cancer-specific survival in univariable analyses, multivariable modelling revealed that tumour stage and grade remained the dominant independent prognostic factors, with nuclear HIF1A not providing additional risk stratification after adjustment. These observations are consistent with the contribution of HIF signalling to aggressive tumour biology, although its prognostic significance beyond established clinicopathological factors remains unresolved in this cohort. TCGA analysis provided a complementary transcriptomic context but should not be interpreted as an independent validation of tissue-based clinical findings. The identification of nuclear HIF1A and EPAS1/HIF2A as tumour-specific and biologically active features of ccRCC provided a rationale for the subsequent functional interrogation of the HIF axis as a therapeutic target. Beyond prognostication, the central aim of this study was to explore the therapeutic vulnerability of the HIF axis, particularly in the context of resistance to VEGFR-targeted therapy [7,8]. Our in vitro experiments demonstrated that pharmacological modulation of HIF signalling produces profoundly different biological outcomes, depending on the mechanism of action of the inhibitor. Among the tested compounds, GN44028 emerged as the most potent and consistent agent, exerting strong antiproliferative effects in both VHL-mutant (786-O) and VHL-wild-type (Caki-1) ccRCC cell lines. Importantly, comparable or lower IC_50_ values were also observed in non-malignant HK-2 cells, indicating that GN44028 does not exhibit a classical therapeutic window at the level of cell viability in the current study. Nevertheless, the biological response differed between malignant and non-malignant cells: in cancer cell lines, GN44028 was associated with an increased sub-G1 fraction, whereas in HK-2 cells, the dominant effect was G2/M arrest. These findings suggest qualitatively distinct cellular outcomes following HIF pathway suppression but do not support claims of cancer-selective cytotoxicity. The translational relevance of in vitro IC_50_ values and phenotypic effects remains limited, as these measurements do not fully capture systemic pharmacokinetics, tumour microenvironment, and potential off-target toxicity in vivo. Therefore, the observed effects should be interpreted as indicative of biological activity, rather than direct predictors of clinical efficacy or safety. Functional assays further revealed that GN44028 markedly reduced wound closure, particularly in the Caki-1 cell line, where wound closure was almost completely inhibited [23]. However, given the concomitant antiproliferative effects and cell cycle alterations observed at the tested concentrations, these findings should be interpreted as reflecting the combined effects on cell growth and wound closure dynamics rather than direct inhibition of cell migration [24].

In contrast, KC7F2 and FM19G11 displayed weaker and less consistent effects, and in some contexts, were associated with greater wound closure in Caki-1 cells. These findings highlight that not all HIF-targeting compounds are biologically equivalent and underscore the importance of mechanistic specificity in evaluating HIF-directed interventions. Because the present wound closure assay does not disentangle migration from proliferation-related effects, more specific approaches, such as transwell or matrix-based invasion assays, are required to determine whether any of these compounds directly affect cell migration or invasion. At the transcriptional level, qPCR analysis provided mechanistic insights into these phenotypic effects. These observations are consistent with experimental evidence demonstrating that sustained HIF1A activity promotes metabolic reprogramming, epithelial–mesenchymal transition, and acquisition of aggressive tumour phenotypes across multiple cancer types. Recent studies have further implicated HIF1A in immune evasion mechanisms in ccRCC, linking its nuclear activity to PD-L1 expression and resistance to immune-mediated cytotoxicity [25,26,27]. Although not all genes included in the analysed signature are equally well characterised in renal cell carcinoma, they converge on key biological processes relevant to tumour aggressiveness, including angiogenesis (*VEGFA*, *EDN1*, and *CA9*), epithelial–mesenchymal transition (*VIM and MMP2*), metabolic reprogramming (*ENO1*), and stemness-associated programs (*POU5F1*) [22]. Their coordinated regulation by HIF signalling, as demonstrated both experimentally and in TCGA data, supports their inclusion as biologically meaningful components of a hypoxia-driven transcriptional network rather than as isolated biomarkers. GN44028 induced broad and deep suppression of the hypoxia-associated transcriptional program, including *HIF1A*, *EPAS1/HIF2A*, *VEGFA*, *VI*M, and *POU5F1*, in both the ccRCC models. This contrasts sharply with the effects of sunitinib, which failed to suppress core HIF targets and was associated with treatment-induced upregulation of *EPAS1/HIF2A*, *VEGFA*, and *EDN1*, particularly in 786-O cells. Sunitinib treatment was associated with increased expression of EPAS1/HIF2A and selected HIF-related transcripts in 786-O and Caki-1 cells, respectively. Importantly, the present data do not establish a causality between these transcriptional changes and clinically relevant resistance and should be interpreted as exploratory observations. This observation is consistent with our previous report, which showed that sunitinib upregulates *VEGFA* and *c-myc* genes in 786-O and ACHN RCC cell lines [9,15]. These observations are consistent with previously reported associations between VEGFR-targeted therapy and activation of hypoxia-related transcriptional programs and may reflect an adaptive cellular response to VEGFR blockade rather than direct evidence of a resistance mechanism [28,29].

GN44028 was used as a mechanistic tool compound to investigate the therapeutic consequences of broad HIF pathway suppression rather than as a clinically optimised drug candidate. Importantly, GN44028 has been previously characterised as an inhibitor of HIF-1α transcriptional activity, acting at the level of functional transcriptional output, rather than mRNA expression or protein stability [30]. In this context, the coordinated downregulation of canonical HIF target genes observed in our experiments can be interpreted as a functional readout of HIF pathway inhibition. The lack of a clear therapeutic window in viability assays further supports this hypothesis. The observed antiproliferative effects in non-malignant HK-2 cells further underscore that GN44028 should be regarded as an experimental probe rather than a selective anticancer agent at this stage. Accordingly, the purpose of GN44028 in this study was not to propose an immediate drug candidate but to experimentally test whether broad suppression of hypoxia-driven transcription produces a distinct biological response compared with indirect anti-angiogenic treatment. The clinical relevance of these findings is partially supported by TCGA-based analyses restricted to patients with advanced (stages III–IV) ccRCC. Importantly, the TCGA-based analyses were not intended to establish a clinically actionable predictive model or directly validate the clinical cohort but rather to contextualise the experimental findings within an independent transcriptomic dataset [31,32]. Moreover, TCGA analysis should be interpreted as a complementary dataset rather than a direct validation of the clinical cohort. Differences in endpoint definition (overall survival in TCGA vs. cancer-specific survival in our cohort), patient selection criteria, and expression stratification strategies introduce systematic heterogeneity that limits direct comparability of the results. Nevertheless, the observed directional consistency across the datasets supports the broader biological relevance of HIF-associated transcriptional patterns. Prospective clinical validation, including correlation with responses to immune checkpoint inhibitors or VEGFR-targeted therapies, is required to assess the translational utility of these findings. In this cohort, high *HIF1A* expression was consistently associated with worse overall survival, whereas reduced expression of *EPAS1/HIF2A*, *CA9*, and *EDN1* identified patients with particularly unfavourable outcomes. These results emphasise that HIF1A and EPAS1/HIF2A play non-redundant and, in some contexts, opposing roles in advanced diseases. An important limitation of the present study is the apparent discrepancy between clinical IHC findings and TCGA-based transcriptomic analysis for EPAS1/HIF2A. While increased nuclear EPAS1/HIF2A expression was associated with worse cancer-specific survival in our cohort, lower EPAS1/HIF2A mRNA levels in TCGA were associated with worse overall survival in the TCGA cohort. This difference likely reflects the fundamentally distinct biological layers captured by these methods. TCGA data represent bulk transcriptomic measurements derived from heterogeneous tumour tissue, including stromal and immune components, whereas our IHC analysis specifically evaluates the nuclear localisation of EPAS1/HIF2A within tumour cells, corresponding to its transcriptionally active state. Moreover, differences in clinical endpoints (cancer-specific vs. overall survival) and the restriction of TCGA analysis to advanced-stage disease may further contribute to the observed divergence. Therefore, these findings should not be interpreted as directly conflicting but rather as complementary, highlighting the complexity of HIF pathway regulation and the importance of considering both the expression level and subcellular localisation when interpreting its biological and clinical significance. Accordingly, the TCGA results provide convergent but non-confirmatory support for the biological relevance of the observed HIF-associated transcriptional patterns. Correlation and network analyses further positioned HIF1A as a central node linking mesenchymal markers (VIM), extracellular matrix remodelling enzymes (MMP2), and angiogenic mediators, reinforcing its role as a master regulator of aggressive tumour phenotypes. Network topology analyses further emphasise that HIF1A and EPAS1/HIF2A occupy distinct regulatory positions within the hypoxia-driven transcriptome, supporting context-dependent and non-redundant biological functions in advanced diseases. The divergent prognostic associations of HIF1A and EPAS1/HIF2A observed in advanced ccRCC underscore the context-dependent roles of individual HIF isoforms. This observation is consistent with emerging clinical data demonstrating selective therapeutic benefits from EPAS1/HIF2A inhibition, suggesting that complete suppression of hypoxia-driven transcriptional programs may be required to overcome resistance mechanisms.

Importantly, the apparent protective association of higher *EPAS1/HIF2A* expression in advanced ccRCC may help explain the complex and sometimes contradictory clinical responses to selective EPAS1/HIF2A inhibition. These findings align with emerging clinical and preclinical evidence indicating that selective EPAS1/HIF2A inhibition, although effective in specific contexts, may be insufficient to suppress the broader hypoxia-driven transcriptional landscape in advanced ccRCC. Mechanistic studies have suggested that EPAS1/HIF2A signalling may contribute to resistance to VEGFR-targeted therapies, reinforcing the rationale for therapeutic strategies that simultaneously target multiple layers of HIF signalling [33,34,35]. While belzutifan has demonstrated clinical efficacy [11,13,14], our data suggest that global suppression of the hypoxic transcriptional program, rather than isolated EPAS1/HIF2A blockade, may be required to overcome adaptive resistance mechanisms. In this context, the transcriptional effects of GN44028 more closely resemble a broad suppression of hypoxia-driven oncogenic signalling than that elicited by sunitinib.

This study has several limitations. Analysis of patient material was restricted to IHC due to the use of archival FFPE specimens, precluding parallel protein-level validation by Western blotting or proteomics. Similarly, protein-level confirmation of HIF modulation in cell lines was not performed in the current study and will be addressed in ongoing studies. The current study was not designed to establish therapeutic selectivity across a broader range of non-malignant cell types. The qPCR analysis was based on two independent biological replicates, which represent a minimal level of replication for multi-gene expression studies and may increase susceptibility to variability and effect size inflation. Although technical triplicates were included to ensure assay-level consistency, the transcriptional findings should be interpreted as exploratory and hypothesis-generating. Concordance with complementary TCGA datasets and tissue-based IHC observations partially supports their biological relevance; however, validation in larger experimental series is required. Moreover, the present study did not stratify TCGA-ccRCC cases according to *VHL*, *PBRM1*, or *BAP1* mutation status [36]. These key genetic alterations influence HIF pathway activity and are associated with distinct transcriptional programs and tumour phenotypes. In particular, VHL loss leads to the stabilisation of HIF-α subunits, whereas PBRM1 and BAP1 mutations have been linked to divergent biological behaviours and clinical outcomes in ccRCC. While these alterations are known modulators of HIF signalling [36], our analytical focus was on the downstream transcriptional and phenotypic consequences of HIF pathway activation. Therefore, genotype-specific differences may contribute to the variability in the observed transcriptional and phenotypic responses. Future studies integrating mutational, epigenetic, and transcriptional layers are necessary to dissect the genotype-specific regulation of HIF-driven programs in ccRCC. The lack of in vivo validation represents an additional limitation; the present study is limited to in vitro and ex vivo analyses and does not include the in vivo validation of GN44028. Future studies are required to evaluate the therapeutic potential, pharmacokinetics, and tumour selectivity of GN44028 in appropriate animal models. Such investigations are essential to determine the translational relevance of HIF pathway modulation in ccRCC. Importantly, these limitations are partially mitigated by the concordance between nuclear HIF protein localisation in tissue specimens, transcriptional signatures observed in cell lines, and independent TCGA transcriptomic analyses. This convergence across orthogonal datasets supports the biological relevance of the observed HIF-dependent effects; however, validation in larger experimental and in vivo studies is required.

In summary, this study links nuclear HIF activation and HIF-associated transcriptional programs with the aggressive biological features of ccRCC across tissue, cell line, and TCGA-based analyses. GN44028 exhibited broad transcriptional and phenotypic activity in vitro, whereas sunitinib was associated with increased expression of selected HIF-related transcripts under the tested conditions. These observations are exploratory and hypothesis-generating and do not establish causal links between HIF modulation, therapeutic resistance, or clinical benefit. They support further experimental investigation of broader HIF-pathway modulation in advanced ccRCC.

## 4. Materials and Methods

### 4.1. Patients and Samples

ccRCC tumour tissues and corresponding morphologically unchanged kidney samples from each patient were obtained during radical nephrectomy from 40 patients who underwent radical nephrectomy at the Department of Urology, Medical University of Gdańsk (Gdansk, Poland). Samples were collected over a 4-year period from 2017 to 2020. The exclusion criteria were as follows: other than ccRCC histological subtypes of renal cancer, multifocal and/or bilateral kidney tumours, and Von Hippel–Lindau disease. The study was approved by the Independent Bioethics Committee for Scientific Research at the Medical University of Gdańsk (decision nos. NKEBN/4/2011 and NKBBN/370/2016). Written informed consent was obtained from each patient before surgery [15].

Tissue samples for histopathological assessment and immunohistochemistry (IHC) staining were placed in test tubes filled with 5 volumes of 4% buffered formalin (POCh, Poland). Tumour samples (*n* = 40) were included in the analysis if >60% of the cells in the respective histological sections presented characteristic features of ccRCC. Non-cancerous kidney tissue samples (*n* = 40) were included as matched controls. These samples were obtained from morphologically normal renal parenchyma collected from the surgical margin of the same nephrectomy specimens as the tumour tissue. Thus, each tumour sample had a corresponding paired non-cancerous tissue sample from the same patient. All non-cancerous tissues were evaluated by a pathologist to confirm the absence of neoplastic changes [37,38]. Materials that did not fulfil both conditions were excluded from the study. Tumour stage was assessed according to the Union for International Cancer Control TNM 8th staging edition of the RCC guidelines [17]. The degree of tumour malignancy was determined using the World Health Organization/International Society of Urological Pathology grading system [18]. The tissues were fixed in 4% buffered formalin and stored at 4 °C until further analysis. IHC staining preparation included dehydration, paraffin embedding, and cutting into 5 µm thick sections.

### 4.2. Immunohistochemistry

IHC staining was performed at the Department of Anatomy and Histology, University of Warmia and Mazury, Olsztyn. Immunohistochemical analysis was performed as previously described by Kieżun et al. with modifications [15,39]. The sections were subjected to an antigen retrieval procedure by microwaving for 12 min in Retrieval Solution Buffer, pH 6.0 (Leica Microsystems, Wetzlar, Germany), and incubated with 3% H_2_O_2_ in methanol for 10 min to block endogenous peroxidase activity. Next, non-specific binding sites were blocked with 2.5% normal horse serum (Vector Laboratories, Burlingame, CA, USA) for 30 min. The sections were incubated overnight at 4 °C with rabbit anti-human antibodies against HIF1A (polyclonal, Cat. No. SAB1306172, Sigma-Aldrich; Merck KGaA, Darmstadt, Germany) and HIF2A (monoclonal, Cat. No. SAB2702342, Sigma-Aldrich; Merck KGaA), diluted 1:800 and 1:2000 in PBS. The next day, the sections were incubated with secondary antibodies (ImmPRESS Universal reagent Anti-Mouse/Rabbit Ig, Vector Laboratories, Burlingame, CA, USA) for 30 min [40]. The specificity of immunohistochemical staining was checked by omitting the primary antibody and replacing it with rabbit serum. To visualise the immunoreaction, the sections were stained with DAB (Dako, Carpinteria, CA, USA), counterstained with haematoxylin (Sigma-Aldrich, Merck KGaA, Darmstadt, Germany), dehydrated in an ethanol series, rinsed in xylene, and mounted in DPX (Sigma-Aldrich, St. Louis, MO, USA). The labelled tissues were photographed using an XC-50 camera (Olympus Corporation, Tokyo, Japan) mounted on a light microscope (BX-41; Olympus Corporation). Concomitantly with IHC, H&E staining was performed to assess tissue morphology after 10 sections of slides were used for IHC [15].

### 4.3. Evaluation of IHC Results

We used two different approaches to assess the immunoexpression of HIF1A and HIF2A proteins in the specimens: the IRS method [41] and nuclear counting [20,21]. The assessment included three randomly selected parts of the slide, magnified 200×. The evaluation was performed by three independent histologists (PMW, AK-Ch, and MK-W) who were blinded to the patients’ clinical data. The IRS scale is based on the percentage of cells showing positive reaction, 1 point: 1–10% cells, 2-points: 11–50%, 3 points: 51–80%, and 4 points: over 80% cells with positive reaction, as well as reaction intensity (0: no reaction, 1: low-intensity reaction, 2: moderate-intensity reaction, and 3: intense reaction). The final score ranged from 0 to 12 points and depended on both parameters, the percentage of positive cells and the intensity of the reaction [15,41]. The nuclear H-score method was also used because of the role of HIF1A and HIF2A as transcription factors: H-score = (1 × % of cells with weak intensity in nucleus) + (2 × % of cells with moderate intensity in nucleus) + (3 × % of cells with strong intensity in nucleus); yielding results ranging from 0 to 300 [20,21].

### 4.4. Cell Cultures

786-O (cRL1932TM), Caki-1 (McCoy) human renal cell carcinoma lines, and HK-2 (cRL2190TM) human proximal kidney tubule-derived cell lines were obtained from the American Type Culture Collection (ATCC, Manassas, VA, USA). The 786-O and Caki-1 cells were cultured in RPMI-1640 medium (Sigma-Aldrich; Merck KGaA) and Eagle’s Minimum Essential Medium (MEM; Sigma-Aldrich; Merck KGaA), respectively. Both media were supplemented with 10% FBS (Sigma-Aldrich; Merck KGaA) and 1% penicillin-streptomycin (Sigma-Aldrich; Merck KGaA), with MEM also containing an additional 2% L-glutamine. HK-2 cells were cultured in keratinocyte serum-free medium (Gibco; Thermo Fisher Scientific, Inc., Waltham, MA, USA). Cell cultures were conducted in a sterile MCU-170 incubator (Panasonic, Osaka, Japan) at 37 °C with 5% CO_2_ and routinely tested for Mycoplasma presence (MycoAlert PLUS, Lonza, Walkersville, MD, USA) every 2 months.

### 4.5. Reagents

KC7F2 ((C_16_H_16_Cl_4_N_2_O_4_S_4_), abbreviation: KC) is a small-molecule inhibitor targeting HIF-1α, showing potential in suppressing tumour growth in cancers like breast and colon cancer by disrupting hypoxia-induced pathways; however, its clinical use is limited by poor solubility and bioavailability [42]. GN44028 ((C_18_H_15_N_3_O_2_), abbreviated as GN) is a small-molecule inhibitor reported to suppress HIF-1α transcriptional activity without affecting its expression or stability [30]. Preclinical studies have indicated its efficacy in various solid tumours, although it remains largely investigational [43,44,45]. FM19G11 ((C_23_H_17_N_3_O_8_), abbreviated as FM) inhibits HIF1A and HIF2A proteins, reducing tumour progression in hypoxic environments, with preclinical evidence suggesting its potential in cancers such as glioblastoma; however, it is not yet approved for clinical use [46]. Sunitinib, a multi-targeted TKI, is a first-line drug for advanced ccRCC and other cancers. It blocks VEGF and PDGF receptors to inhibit tumour angiogenesis and growth, although resistance can develop [16,47]. All reagents were purchased from Sigma-Aldrich; Merck KGaA and dissolved in DMSO (Sigma-Aldrich; Merck KGaA) according to the manufacturer’s instructions, with the final concentration of DMSO in the cell culture medium not exceeding 1%.

### 4.6. Cell Viability Assay

The cytotoxicity of KC, GN, FM, and sunitinib was evaluated using a Sulforhodamine B (SRB) assay [9,48]. In summary, the cells were seeded into 96-well plates at the following density: 786-O, 5 × 10^3^ cells/well; Caki-1, 5 × 10^3^ cells/well; and HK-2, 5 × 10^3^ cells/well. The cells were then incubated for 24 h in their respective media with no reagents added. After incubation, varying concentrations of the inhibitors (Sunitinib, 0–20 μM; KC, 0–60 μM; GN, 0–80 μM; FM, 0–60 μM) were added to the wells. After 24, 48, or 72 h of incubation with the inhibitors, the cells were fixed by adding trichloroacetic acid (TCA) to a final concentration of 10% and incubated for 1 h at 4 °C. The plates were then washed with deionised water, drained for approximately 30 min, and placed in an incubator for 10 min. Each well was stained with 100 μL of SRB dissolved in 1% acetic acid for 15 min at room temperature. The plates were then rinsed with 1% acetic acid to remove excess dye and dried overnight. Finally, 150 μL of TRIS buffer (10 mM, pH 10.5) was added to each well, and the plates were gently shaken to dissolve the stain. Absorbance was measured at 540 nm with a reference wavelength of 630 nm using an Epoch UV universal microplate reader (BioTek Instruments, Inc., Winooski, VT, USA). The results were recalculated, and values were obtained for the concentrations of the respective compounds at which 50% of the cells survived (IC_50_). Blank subtraction was calculated first, followed by normalisation to the vehicle control (DMSO = 1 or 100%). Experiments were performed in six independent biological replicates, each measured in triplicates.

### 4.7. Cell Cycle Analysis

786-O, Caki-1, and HK-2 cells were seeded at a density of 2 × 10^5^ cells/well in 6-well plates. After 24 h of incubation to allow cell attachment, the cells were treated with KC, GN, FM, and sunitinib at their previously determined half-maximal inhibitory concentrations (IC_50_), calculated from the previous step. After 24 h of treatment, cells were harvested, washed twice with phosphate-buffered saline (PBS; Sigma-Aldrich, Merck KGaA), and fixed in 70% ice-cold ethanol for 48 h at 4 °C. Prior to flow cytometry, fixed cells were washed with PBS and stained for 30 min at 37 °C in the dark with a propidium iodide (PI) staining solution containing RNase A (100 µg/mL) and propidium iodide (50 µg/mL) (Sigma-Aldrich, Merck KGaA). Cellular DNA content was measured by flow cytometry (FACSCalibur; Becton Dickinson, Franklin Lakes, NJ, USA) using an excitation wavelength of 536 nm and emission detection at 617 nm. The number of analysed samples per condition ranged from *n* = 3 to *n* = 19, reflecting the exclusion of samples with insufficient event counts or suboptimal DNA content histograms after quality control. Data were analysed using CellQuest Pro software (ver. 5.1, BD Biosciences, San Jose, CA, USA), and the results were expressed as the percentage of cells in sub-G1 (apoptotic), G0/G1, S, and G2/M phases of the cell cycle. Each experiment consisted of at least three independent biological replicates, while technical replicates varied depending on cytometric quality control and event count thresholds.

### 4.8. qPCR Analysis of mRNA Levels in Cultured Cells

A total of 2 × 10^5^ cells/well were seeded in 6-well plates and incubated for 24 h in the appropriate medium. Afterward, the cells were treated with inhibitors at the IC_50_ for 72 h. Total RNA was extracted from the collected samples using the ExtractMe Total RNA kit (Blirt, Inc.; Qiagen N. V., Gdansk, Poland). The extracted RNA was dissolved in 30 μL of REB buffer. RNA quantity and quality were assessed using a spectrophotometer (Nanodrop ND 1000; Thermo Fisher Scientific, Inc.). The RNA samples were stored at −80 °C until further analysis. One microgram of RNA was reverse transcribed using EURx smART Combo First Strand cDNA Synthesis Kit (Euryx, Gdansk, Poland) in a total volume of 20 μL. The reaction was performed according to the manufacturer’s protocol (50 °C, 1 h; 85 °C, 5 min; 4 °C, 5 min). cDNA samples were stored at −20 °C until further analysis.

### 4.9. Assessment of Gene mRNA Levels in Cell Lines

mRNA assessment was performed using the qPCR technique. Primer sequences were designed using the Primer-BLAST software (https://www.ncbi.nlm.nih.gov/tools/primer-blast/, accessed on 8 April 2026). Primer sequences, NCBI mRNA reference number, and qPCR variable temperature of annealing, as well as experimentally established reaction conditions, are presented in Table 6. Each experiment was performed using two independent biological replicates, each analysed in technical triplicates. The measurements were performed using 1.5 μL 4×-diluted cDNA, primers with final concentration of 300 nM, and SG qPCR Master Mix (Euryx) chemistry in a total volume of 15 μL. The reaction was conducted on a separate PCR plate (4titude, Ltd., Wotton-under-Edge, UK) for each gene with a negative control (water instead of cDNA) and 10× diluted pooled cDNA as a precision control. A StepOne Plus apparatus with StepOne Plus software ver. 2.3 (Applied Biosystems; Thermo Fisher Scientific, Inc., Waltham, MA, USA) was used for the amplification process and data analysis. Geometric mean of Ct (threshold cycle) values for each gene were normalized to the reference gene [glucuronidase β (GUSB)], according to our previous normalization study on ccRCC [49], using Livak’s equation [50]: x = 2^ΔCt^, where x stands for expression of gene y and ΔCt = (Ct GUSB − Ct gene Y). The following temp-time profile was experimentally established [9]: 95 °C, 3 min; 40 × (95 °C, 5 s; annealing temperature (Table 6), 10 s; 72 °C, 12 s; 77 °C, 10 s—sample reading); melting curve: 95 °C, 15 s; 60 °C, 1 min; 60 °C → 95 °C reading every 0.3 °C. Obtained raw expression data for each tumour sample were calibrated to average expression data in control cell line with vehicle (DMSO; fold change; control sample = 1).

### 4.10. Wound Closure Assay

The 786-O and Caki-1 cells were seeded in 8-well plates (MoBiTec Molecular Biology, Göttingen, Germany) at the density of 3 × 10^4^ cells/well. Cells were incubated for 48 h in RPMI-1640 and Eagle’s MEM medium, respectively. Both media were supplemented with 10% FBS and 1% penicillin-streptomycin. MEM was additionally supplemented with 2% L-glutamine. Confluent cell cultures were then scratched using a sterile tip and incubated with KC, GN, FM, and sunitinib at IC25 concentration.

Cell wound closure was monitored by live-cell imaging system (Olympus cellVivo IX83). Images were recorded every 30 min as a time-lapse movie using cellSense Dimension Desktop 4.3 and TruAI software (ver. v2.5.3, Olympus, Tokyo, Japan) [51]. The software automatically recognized and marked individual cells, and the percentage of wound closure was calculated relative to the initial wound size. Each experiment was performed in six technical replicates. For quantitative analysis, wound closure was evaluated over 0–20 h in 786-O cells and 0–48 h in Caki-1 cells, corresponding to complete gap closure in control cultures. Because wound closure reflects both migration and proliferation, the assay was not interpreted as a direct measure of migratory capacity.

### 4.11. Statistical Analysis

Statistical analysis was performed according to our previous studies using GraphPad Prism ver. 6.07 (GraphPad Software, Inc., San Diego, CA, USA), Statistics ver. 13.3 (StatSoft Ltd., Hamburg, Germany) software [9,15,16], and R (v4.3.3). First, data normality was checked using the Shapiro–Wilk test. However, since most data for clinical samples did not pass it, the following non-parametric tests were applied: Mann–Whitney U for two groups, Wilcoxon signed-rank if samples were paired, and Spearman’s correlation. Outliers were identified using the ROUT test. Associations between categorical variables were tested using Fisher’s exact test. The receiver operating characteristic (ROC) curve was employed to evaluate the performance of the classification model. Results from ROC analysis were utilized to find cutoff values in cancer tissues using Youden’s index [52,53,54]. Similar ROC-based approaches have been applied previously to immunohistochemical assessment of HIF-2α in ccRCC, demonstrating that quantitative thresholding of HIF expression can reliably distinguish biologically relevant expression states [19]. Data represent independent biological experiments with technical replicates (as specified for each assay). The log2FC vs. DMSO comparison of mRNA levels for a particular gene was used for the determination of upregulation and downregulation of a given gene in cancer cell line. For outcome analysis of patients, Kaplan–Meier survival tests with log-rank (Mantel–Cox) tests were applied, with data acquired for cancer-specific survival (CSS). Cox proportional hazards regression was applied in a two-step approach: univariable analysis (first step) followed by multivariable analysis (second step) [16,55]. Due to the limited number of events (noe = 20) and low events-per-variable ratio (EPV ≈ 1.8) in the full model including all 11 covariates, LASSO-penalized Cox regression was used to select a parsimonious and stable multivariable model [56]. Model stability was assessed via bootstrap resampling (B = 10,000) [57]. The results from cellular assays were analysed using unpaired Student’s t-test (for two-group comparisons) or one-way ANOVA followed by Dunnett’s post hoc test when comparing multiple treatment groups to a single control (DMSO); two-way ANOVA with Tukey’s multiple comparison test was used where appropriate (e.g., time × treatment interactions). A two-sided *p* < 0.05 was considered statistically significant. Unless otherwise stated, all in vitro experiments were performed in at least three independent biological replicates (separate cell cultures initiated on different days), each measured in multiple technical replicates as specified for individual assays. Reported n values reflect the total number of biological observations pooled across experiments. Variability in n between assays (e.g., cell cycle analysis) results from differences in cell line-specific viability, treatment response, and quality-control filtering during flow cytometric acquisition.

### 4.12. TCGA Analysis; Survival Analysis

Unstranded transcriptome expression data for the TCGA-ccRCC cohort was downloaded from the GDC portal using the R package TCGAbiolinks (v2.30.0, https://doi.org/10.1093/nar/gkv1507). Gene expression values were normalized using the Variance Stabilizing Transformation (VST) method from the DESeq2 package (v1.42.0, https://doi.org/10.1186/s13059-014-0550-8). Curated survival data were obtained from Liu et al. (2018) [58]. The initial cohort of 440 TCGA-ccRCC patients with matching survival and expression data was refined by excluding eight cases later reclassified as non-ccRCC (https://doi.org/10.1016/j.celrep.2021.110190). The remaining cohort was then filtered to include only patients with advanced disease (pathological stages III and IV), resulting in a final group of 185 patients for analysis. TCGA-ccRCC cases were restricted to advanced-stage tumours (stage III–IV) to increase biological homogeneity and event rates for survival analysis. No attempt was made to directly match TCGA patients to the clinical cohort. Gene expression levels were stratified using terciles of variance-stabilized (VST) normalized counts to allow balanced group comparisons. Survival analyses were conducted using the survival package (v3.8, https://CRAN.R-project.org/package=survival, accessed on 8 April 2026). The date of diagnosis was set as time zero. The endpoint was defined as the date of event (death for OS), with data censored at 5 years post-diagnosis. For log-rank analysis, patients were stratified into terciles based on the expression of the gene of interest. Hazard ratios (HRs) and corresponding confidence intervals were calculated using Cox proportional-hazards regression. All survival analyses were performed using R (v4.3.3). Correlation analysis was performed using VST-normalized gene expression data from advanced-stage patients. Spearman’s rank correlation coefficients were calculated using the base stats package. Resulting *p*-values were adjusted for multiple comparisons using the Benjamini–Hochberg method. Plots were generated by the ggplot2 package (v3.5.2), with trends illustrated by locally estimated scatterplot smoothing (LOESS) curves. The correlation network was constructed using the igraph package (v2.0.3, https://doi.org/10.5281/zenodo.7682609). All correlation analysis was performed using R (v4.3.3).

## 5. Conclusions

Our results indicate that nuclear accumulation of HIF1A and EPAS1/HIF2A is associated with adverse clinicopathological features in ccRCC, supporting the biological relevance of active HIF signalling in this disease. However, the clinical analyses are exploratory, and the independent prognostic significance of nuclear HIF activity remains uncertain. Among the tested compounds, GN44028 showed the broadest in vitro effects on cell viability, cell-cycle distribution, wound closure dynamics, and HIF-related gene expression, but the present data do not support selective activity toward malignant cells. Sunitinib was associated with increased expression of selected HIF-related transcripts under the tested conditions. Overall, these findings support further experimental investigation of broader HIF-pathway modulation in advanced ccRCC, while larger clinical cohorts, expanded biological replication, protein-level target engagement studies, and in vivo validation will be required before prognostic or translational conclusions can be drawn.

## Figures and Tables

**Table 1:** Clinicopathological characteristics of the patient cohort (*n* = 40). Data are presented as the number of cases (*n*) and percentage (%), unless otherwise indicated.

Clinical and Pathological Variable		*n* = 40	
	Subgroups	*n*	%
Mean ± SD: 67.35 ± 9.52; range: 46–86	<70	19	47.5
	≥70	21	52.5
Sex	Female	11	27.5
	Male	29	72.5
Tumour size (cm)	≤7 cm	18	45.0
	>7 cm	22	55.0
Tumour location	Left	21	52.5
	Right	19	47.5
WHO/ISUP histological grade	1	3	7.5
	2	11	27.5
	3	14	35.0
	4	12	30.0
Pathological stage (TNM)	I	10	25.0
	II	1	2.5
	III	22	55.0
	IV	7	17.5
Cancer-specific survival status	Alive	20	50.0
	Dead	20	50.0

**Table 2:** Receiver operating characteristic (ROC) analysis of HIF1A and EPAS1/HIF2A nuclear H-score and cytoplasmic IRS for prediction of CSS events in patients with ccRCC (*n* = 40, events = 20).

ROC Analysis	HIF1A Nuclear H-Score	EPAS1/HIF2A Nuclear H-Score	HIF1A Cellular IRS Score	EPAS1/HIF2A Cellular IRS Score
AUC	0.6350	0.6384	0.5072	0.5486
Std. Error	0.0622	0.0615	0.0669	0.0658
95% CI	0.5129 to 0.7571	0.5177 to 0.7592	0.3760 to 0.6384	0.4194 to 0.6777
*p* value	**0.0377**	**0.0331**	0.9125	0.4632
Cutoff value in tumour samples	32.00	62.50	4.50	6.50

AUC, area under the curve; CI, confidence interval. Optimal cut-offs were determined using Youden’s index. Nuclear H-score showed modest but statistically significant discriminatory performance in this cohort, whereas cellular IRS showed no discriminatory value. Bold indicates statistical significance.

**Table 3:** Association between nuclear HIF1A and EPAS1/HIF2A immunoexpression (H-score) and clinicopathological parameters in patients with clear-cell renal cell carcinoma (ccRCC) (*n* = 40).

Patients/Proteins		HIF1A Nuclear H-Score (%)			EPAS1/HIF2A Nuclear H-Score (%)		
*n* = 40	Subgroups (*n*)	↓ 12 (30%)	↑ 28 (70%)	*p*-Value	↓ 15 (37%)	↑ 25 (63%)	*p*-Value
Age (years) Mean ± SD 67.35 ± 9.52 Range: 46–86	<70 *n* = 19	5	14	0.736	5	14	0.2
	≥70 *n* = 21	7	14		10	11	
Sex	Female *n* = 11	3	8	1.000	5	6	0.72
	Male *n* = 29	9	20		10	19	
Tumour size (cm)	≤7 cm *n* = 18	6	12	0.738	6	12	0.747
	>7 cm *n* = 22	6	16		9	13	
Tumour location	Left *n* = 21	6	15	1.000	7	14	0.745
	Right *n* = 19	6	13		8	11	
WHO/ISUP histological grade	1 + 2 *n* = 14	7	7	**0.07**	8	6	**0.089**
	3 + 4 *n* = 26	5	21		7	19	
TNM stage	Non-metastatic (I + II) *n* = 11	7	4	**0.007**	7	4	**0.065**
	Advanced (III + IV) *n* = 29	5	24		8	21	

All patients had histologically confirmed clear-cell RCC (ccRCC). Cut-offs: HIF1A ≥ 32 and EPAS1/HIF2A ≥ 62.5 (ROC-derived, Youden’s index). Arrows (↓ and ↑) indicate values below and above the predefined cut-off points, respectively (low vs. high expression groups). *p*-values were calculated using two-sided Fisher’s exact test. The bold text indicates statistical significance (*p* < 0.05) or trend (*p* < 0.1).

**Table 4:** Univariable and multivariable Cox proportional hazards analysis of cancer-specific survival in ccRCC patients (*n* = 40, events = 20).

Parameters	Univariable Analysis			Multivariable Analysis (LASSO)		
	HR (95% CI)	χ^2^	*p*-Value	HR (95% CI)	χ^2^	*p*-Value
Tumour stage III + IV vs. I + II	**12.78 (1.70–95.99)**	6.14	**0.013**	**10.82 (1.40–83.20)**	5.52	**0.016**
ISUP grade 3 + 4 vs. 1 + 2	**3.50 (1.14–10.68)**	4.83	**0.028**	**3.12 (1.02–9.55)**	3.89	**0.048**
HIF1A nuclear H-score ↑ vs. ↓	**6.47 (1.49–28.11)**	6.19	**0.013**	2.10 (0.61–7.25)	1.42	0.233
EPAS1/HIF2A nuclear H-score ↑ vs. ↓	**5.71 (1.65–19.77)**	7.55	**0.006**			

HR, hazard ratio; CI, confidence interval; ↑ vs. ↓ were defined by ROC-derived cut-offs (HIF1A ≥ 32; EPAS1/HIF2A ≥ 62.5). Because the full multivariable model was unstable given the limited number of events, a LASSO-penalised Cox model was used for exploratory variable selection. EPAS1/HIF2A was not retained in the penalised model. Bold indicates statistical significance (*p* < 0.05).

**Table 5:** Half-maximal inhibitory concentrations (IC_50_) of HIF inhibitors and sunitinib in the renal cell lines.

Cell Line	IC_50_ Concentration [μM]			
	FM19G11	KC7F2	GN44028	SUNITINIB
HK-2 (normal)	10.98 ± 1.42	10.23 ± 1.31	3.39 ± 0.51	33.53 ± 4.12
786-O (ccRCC, VHL-mut)	22.28 ± 2.87	19.49 ± 2.55	7.99 ± 1.03	12.47 ± 1.61
Caki-1 (ccRCC, VHL-wt)	31.53 ± 3.98	44.61 ± 5.67	5.77 ± 0.74	4.61 ± 0.59

Values represent mean IC_50_ ± SD (µM) from *n* = 6 independent experiments using SRB assay (72 h treatment).

**Table 6:** Details of the qPCR assays used in the study.

Gene	NCBI #	PCR Product Size [bp]	qPCR Annealing Temp [°C]	
*GUSβ*	NM_000181.4	ATGCAGGTGATGGAAGAAGTGGTG	177	57
		AGAGTTGCTCACAAAGGTCACAGG		
*EDN1*	NM_001168319.2	AGGAGCTCCAGAAACAGTCTTA	239	59
		AATTCTCCAAGGCTCTCTTGGA		
*MMP2*	NM_001127891.3	TCTTAGGTGCTTACCTAGCACA	269	59
		GGTGTGTAGCCAATGATCCTGT		
*ENO1*	NM_001201483.4	GAGACCCAGTGGCTAGAAGTTC	224	59
		GTGGGTTCTAAGGCTTACCCT		
*VIM*	NM_003380.5	CGAAAACACCCTGCAATCTTTC	139	59
		CAGCTCCTGGATTTCCTCTTCG		
*CCN2*	NM_001901.4	GGAAGAGAACATTAAGAAGGGCA	331	58
		GTCTCTCACTCTCTGGCTTCAT		
*POU5F1*	NM_001173531.3	AACATCCTTAAACTGGGGGTGA	346	59
		TTTGGCTGAATACCTTCCCAAA		
*CA9*	NM_001216.3	CTGTCTCGCTTGGAAGAAATCG	201	60
		GAGGGTGTGGAGCTGCTTAG		
*VEGFA*	NM_001025366.3	ACATCACCATGCAGATTATGCG	198	60
		CGTACACGCTCCAGGACTTATA		
*VEGFB*	NM_001243733.2	AAGTCCGGATGCAGATCCTC	297	60
		TCTGAAAAGCCATGTGTCACCT		
*HIF1A*	NM_001243084.2	GAAGGTCTAGGAAACTCAAAACCTG	266	60
		TCAATATCCAAATCACCAGCATCC		
*EPAS1/HIF2A*	NM_001430.5	ATGACAGCTGACAAGGAGAAGA	235	60
		ACTCGTTTTCAGAGCAAACTGA		

## Data Availability

The original contributions presented in this study are included in the article. Further enquiries should be directed to the corresponding author. TCGA data were obtained from the Genomic Data Commons (GDC) portal.

## Abbreviations

AUC	Area Under the Curve
CA9	Carbonic Anhydrase IX
CCN2	Cellular Communication Network Factor 2
ccRCC	Clear-Cell Renal Cell Carcinoma
CI	Confidence Interval
CSS	Cancer-Specific Survival
DMSO	Dimethyl Sulfoxide
EDN1	Endothelin 1
EMT	Epithelial–Mesenchymal Transition
ENO1	Enolase 1
EPAS1	Endothelial PAS Domain Protein 1 (Hypoxia-Inducible Factor 2)
FFPE	Formalin-Fixed Paraffin-Embedded
GDC	Genomic Data Commons
GUSB	Glucuronidase Beta
HIF	Hypoxia-Inducible Factor
HIF1A	Hypoxia-Inducible Factor 1 Alpha
HIF2A	Hypoxia-Inducible Factor 2 Alpha
HK-2	Human Kidney-2 Cell Line
HR	Hazard Ratio
IC_50_	Half-Maximal Inhibitory Concentration
IHC	Immunohistochemistry
IRS	Immunoreactive Score
MMP2	Matrix Metalloproteinase 2
mTOR	Mechanistic target of rapamycin
OS	Overall Survival
PBS	Phosphate-Buffered Saline
PD-1	Programmed cell death protein 1
PI	Propidium Iodide
POU5F1	POU Class 5 Homeobox 1
RCC	Renal Cell Carcinoma
ROC	Receiver Operating Characteristic
SRB	Sulforhodamine B
TCGA	The Cancer Genome Atlas
TKI	Tyrosine Kinase Inhibitor
TNM	Tumour–Node–Metastasis
VEGF	Vascular Endothelial Growth Factor
VEGFA	Vascular Endothelial Growth Factor A
VEGFB	Vascular Endothelial Growth Factor B
VEGFR	Vascular Endothelial Growth Factor Receptor
VHL	von Hippel–Lindau
VIM	Vimentin
VST	Variance Stabilizing Transformation
UICC	Union for International Cancer Control
